# Global assessment of coralline algae mineralogy points to high vulnerability of Southwestern Atlantic reefs and rhodolith beds to ocean acidification

**DOI:** 10.1038/s41598-022-13731-y

**Published:** 2022-06-10

**Authors:** Rodrigo Tomazetto de Carvalho, Gustavo Miranda Rocha, Claudia Santiago Karez, Ricardo da Gama Bahia, Renato Crespo Pereira, Alex Cardoso Bastos, Leonardo Tavares Salgado

**Affiliations:** 1 Instituto de Pesquisas Jardim Botânico do Rio de Janeiro, Rua Pacheco Leão, 915, Jardim Botânico, Rio de Janeiro, RJ CEP 22460-030 Brazil; 2 Instituto de Biofísica Carlos Chagas Filho, CCS-Centro de Ciências da Saúde, Cidade Universitária, Rio de Janeiro, RJ 21941-902 Brazil; 3 Departamento de Ecologia e Recursos Naturais, Centro de Ciências Humanas e Naturais, Universidade Federal do Espírito Santo, Av. Fernando Ferrari, 514 Goiabeiras, Vitória, ES CEP 29055-460 Brazil

**Keywords:** Biochemistry, Biogeochemistry, Climate sciences, Ocean sciences, Plant sciences, Climate-change ecology

## Abstract

Coralline algae constitute one of the main groups of highly vulnerable calcified benthic organisms to ocean acidification. Although damaging effects of seawater acidification on the coralline algae skeleton have been widely demonstrated, the susceptibility to dissolution varies according to the Mg^2+^ in the calcite lattice. Even though the Southwest Atlantic Ocean exhibits the world’s largest rhodolith beds, which occupies 20,902 km^2^, there is no information regarding the coralline algae species mineralogy in this area. Here, we provide mineralogical data of twenty-four coralline algae species, examine the similarity in taxonomic groups, spatial occurrence and the vulnerability of these algae to seawater acidification. Mineralogy revealed that coralline algae skeletons were mainly composed of high-Mg calcite (> 70%) with minor presence of aragonite (< 30%) and dolomite (< 3%). There were no similarities between the skeletal mineralogy of taxonomic groups and sampling regions. Remarkably, the mean Mg-substitution of encrusting coralline algae from the Brazilian Shelf was 46.3% higher than global average. Because of the higher mean Mg-substitution in calcite compared with worldwide coralline algae, these algae from Southwest Atlantic Ocean would be highly susceptible to dissolution caused by the expected near-future ocean acidification and will compromise CaCO_3_ net production across the Brazilian Shelf.

## Introduction

Coralline algae are among the most abundant benthic organisms in the marine photic zone, where they grow as crustose and erect forms, either attached to the substrate or forming free-living nodules known as rhodoliths^1,2^. These algae are widely distributed from the tropics to the poles and occur from shallow intertidal zones up to 260 m depths^3^. Coralline algae induce settlement and provide structural complexity and shelter for numerous marine organisms^4^, and often function as major coral reef framework builders^5–7^.

Biomineralization in coralline algae occurs within cell walls, which become completely calcified since their early developmental stages^8^. Crystals formed by coralline algae are mainly calcite with a high degree of Ca^2+^ substitution for Mg^2+^ in the crystal lattice^9^. When Ca^2+^ substitution for Mg^2+^ exceeds 5%, the crystalline polymorph is reclassified as “high-Mg calcite”^10^. This is the case of calcareous algae, since Mg-substitution rate ranges between 9 and 25 wt. %, with most coralline algae containing over 12 wt.% of Mg^11^. Other calcium carbonate polymorphs also occur in coralline algae skeletons. Aragonite is generally found in smaller proportions than high-Mg calcite, but may reach up to 27 wt.% in some coralline algae^11^, while dolomite is overall less important^12^.

The discovery of several CaCO_3_ polymorphs forming coralline algae skeletons and the high degree of Mg-substitution in calcite has boosted interest in its mineralogy to understand how this structure would respond to predicted future changes in seawater conditions, such as temperature rise and pH decrease^2^. The increasing concentration of carbon dioxide (CO_2_) in the atmosphere changes seawater chemistry by decreasing seawater pH and carbonate saturation state (Ω)^11,13,14^. Such changes impair calcification and increase of CaCO_3_ dissolution among coralline algae and corals. Given that high-Mg calcite is the most soluble carbonate form in seawater^13^, coralline algae are expected to be strongly affected by pH decrease.

Calcium carbonate mineralogy has been described for 135 species of red algae, including 108 coralline algae (82 Corallinales, 21 Hapalidiales and 5 Sporolithales)^11^. Fragoso et al*.*^15^ hypothesized that coralline algae possess phylogenetic control over small biomineralogical variations. However, afterward studies did not find a clear relationship between this evolutionary feature and taxonomic characters^11^.

Although the Brazilian Continental Shelf presents the world’s highest non-geniculate coralline algae richness (55 species^16–19^), the largest rhodolith bed (~ 20,900 km^2^) and the highest latitudinal extension of rhodolith beds occurrence (1°N to 23°S), there is no information concerning coralline algae mineralogy from this broad region.

Here, we describe the mineralogy of the coralline algae species of the Brazilian Shelf and their oceanic islands, testing for a possible association between mineralogy and taxonomy. In addition, the results are compared with Crustose Coralline Algae (CCA) mineral composition data from species from other world regions to provide a global-scale overview and a discussion on CCA potential vulnerability to predictable scenarios of ocean acidification.

## Results

### Mineralogy of coralline algae species found in the Brazilian Shelf

High-Mg calcite was the prevalent calcium carbonate polymorph in the cell wall of the coralline algae species analyzed herein, 80% of which with more than 90 wt.% (Table 1).

**Table 1:** Mineralogy (mean percentage ± standard deviation) of coralline algae species collected in several sites along the Brazilian shelf.

Species	Family	Site	% Mg calcite	% aragonite	% dolomite	% Mg in calcite
*Amphiroa* *anastomosans*	Corallinaceae	Espírito Santo	99.1 ± 0.2	0.9 ± 0.2	0	16.7 ± 0.4
*Amphiroa* *brasiliana*	Corallinaceae	Espírito Santo	98.3 ± 0.4	1.7 ± 0.1	0	16.9 ± 0.5
*Amphiroa* *fragilissima*	Corallinaceae	Espírito Santo	97.6 ± 0.3	2.4 ± 0.7	0	18.7 ± 0.2
*Amphiroa* *rigida*	Corallinaceae	Espírito Santo	98.5 ± 0.1	1.5 ± 0.2	0	21.3 ± 0.3
*Corallina* *panizzoi*	Corallinaceae	Espírito Santo	97.8 ± 0.4	2.2 ± 0.4	0	15.6 ± 0.2
*Harveylithon* *rupestre*	Corallinaceae	Saint Peter & Saint Paul Island	87.5 ± 0.5	7.3 ± 0.3	5.2 ± 0.7	32.5 ± 0.7
*Jania* *adhaerens*	Corallinaceae	Espírito Santo	98.7 ± 0.3	1.3 ± 0.2	0	17.6 ± 0.3
*Jania* *cubensis*	Corallinaceae	Espírito Santo	99.7 ± 0.5	0.3 ± 0.1	0	15.9 ± 0.4
*Jania* *subulate*	Corallinaceae	Espírito Santo	99.1 ± 0.2	0.9 ± 0,2	0	18.1 ± 0.3
*Lithophyllum* *attlanticum*	Corallinaceae	Santa Catarina	95.7 ± 0.5	3.5 ± 0.2	0.8 ± 0.2	16.4 ± 0.4
*Lithophyllum* *corallinae*	Corallinaceae	Rio de Janeiro	97.9 ± 0.2	2.1 ± 0.1	0	25.2 ± 0.2
*Lithophyllum* *kaiseri* (as *Lithophyllum* *congestum)*	Corallinaceae	Bahia	98.6 ± 0.2	1.1 ± 0.2	0.3 ± 0.1	15.8 ± 0.5
*Lithophyllum* *margaritae*	Corallinaceae	Santa Catarina	98.4 ± 0.3	1.4 ± 0.2	0.2 ± 0.1	13.8 ± 0.4
*Lithophyllum* *stictaeforme*	Corallinaceae	Bahia	96.8 ± 0.3	2.5 ± 0.3	0.7 ± 0.1	17.8 ± 0.6
*Neogoniolithon* sp.	Corallinaceae	Bahia	99.3 ± 0.3	0.7 ± 0.1	0	25.7 ± 0.4
*Pneophyllum* *conicum*	Corallinaceae	Bahia	90.4 ± 0.3	6.7 ± 0.4	2.9 ± 0.3	25.7 ± 0.5
*Porolithon* *onkodes*	Corallinaceae	Bahia	92.5 ± 0.5	7.1 ± 0.1	0.4 ± 0.2	24.2 ± 0.3
*Lithothamnion* *crispatum*	Hapalidiaceae	ES/BA/AM/Trindade Island	82.3 ± 4.1	16.9 ± 4.2	0.8 ± 0.2	18.9 ± 2.6
*Lithothamnion* *muelleri*	Hapalidiaceae	Bahia	89.7 ± 0.4	10.3 ± 0.3	0	14.5 ± 0.6
*Melyvonnea* *erubescens*	Hapalidiaceae	BA/ES	94.8 ± 0.9	4.3 ± 0.4	0.9 ± 0.3	21.6 ± 0.8
*Sporolithon* *episporum*	Sporolithaceae	Bahia	95.5 ± 0.3	4.5 ± 0.2	0	19.9 ± 0.5
*Sporolithon* *amadoi*	Sporolithaceae	Fernando de Noronha Island	97.8 ± 0.3	0.6 ± 0.1	1.6 ± 0.4	24.5 ± 0.3
*Sporolithon* sp.	Sporolithaceae	Rio de Janeiro	81.7 ± 0.7	18.3 ± 0.2	0	23.8 ± 0.5
*Sporolithon* *yoneshigueae*	Sporolithaceae	Bahia	69.8 ± 0.4	30.2 ± 0.4	0	28.9 ± 0.9

*BA* Bahia State, *ES* Espírito Santo State, *AM* Amazonas State.

The highest and lowest amounts were recorded in *Jania* *cubensis* (99.7 wt.%) and *Sporolithon* *yoneshigueae* (69.8 wt.%)*,* respectively (Table 1). Aragonite was less abundant but was also found in all species, with the highest and lowest amounts found in *S.* *yoneshigueae* (30.2 wt.%) and *J.* *cubensis* (0.3 wt.%), respectively (Table 1). Dolomite was found in nine species and always in low contents (< 5.2 wt.%) (Table 1). Average Mg substitution was 21.1 (± 5.2) wt.% and was highest (32.5 wt.%) in *H.* *rupestre* and lowest (13.8 wt.%) in *Lythophyllum* *margaritae* (Table 1). In the Abrolhos region, the highest Mg-substitution (25.7 wt.%) was found in *Neogoniolithon* *sp.* and *Pneophyllum* *conicum*, the latter being the major reef framework builder^20^.

### Brazilian Shelf coralline algae skeletal mineralogy versus taxonomy and sampling regions

High-Mg calcite in Corallinaceae was 96.7 ± 3.4 wt.%, which was significantly higher than in Sporolithaceae, 86.2 ± 6.5 wt.% (Kruskal–Wallis, p < 0.01; Table 2). However, this content found in Corallinaceae was not different from that mean high-Mg calcite content found in the members of Hapalidiaceae, 88.9 ± 3.6 wt.% (Kruskal–Wallis, p = 0.061; Table 2). There was no difference in high-Mg calcite content between Hapalidiaceae and Sporolithaceae (Kruskal–Wallis, p = 0.14; Table 2).

**Table 2:** Mineralogy (mean ± standard deviation) of coralline algae families Corallinaceae, Hapalidiaceae and Sporolithaceae.

Family	Corallinaceae	Hapalidiaceae	Sporolithaceae
% high Mg-calcite	96.7 ± 3.4^A^	88.9 ± 3.6^AB^	86.2 ± 6.5^B^
% Aragonite	2.7 ± 1.5^A^	10.5 ± 1.4^B^	13.4 ± 3.5^B^
% Dolomite	0.6 ± 0.6^A^	0.6 ± 0.2^A^	0.4 ± 0.4^A^
% Mg in calcite	19.9 ± 3.5^A^	18.57 ± 4.7^A^	24.28 ± 1.9^A^

Different letters are used to indicate statistically differences (p < 0.05) of percentage of CaCO_3_ polymorphs obtained through post-hoc Dunn’s test after the Kruskal–Wallis analysis.

In relation to aragonite, Corallinaceae presented the lowest amount of this polymorph (2.7 ± 1.5 wt.%) compared to Hapalidiaceae (11.6 ± 1.4 wt.%) and Sporolithaceae (13.4 ± 3.5 wt.%; Kruskal–Wallis, p = 0.01). There was no difference between Hapalidiaceae and Sporolithaceae (Kruskal–Wallis, p = 0.507; Table 2). In addition, dolomite contents in the three families were not different (Kruskal–Wallis, p = 0.451). There was no difference either in the mean Mg-substitution at calcite lattice between the three families (Kruskal–Wallis, p = 0.18; Table 2). SIMPROF test showed no grouping identified as significantly different from each other at the 0.05 confidence level (Fig. S1).

When cluster analysis was performed with the mineralogical data of the sixteen different encrusting species, there was no grouping with taxonomic classification, and there was none with sample location (Fig. S2). Different families were grouped as a function of their mineralogical properties, although we observed one exception, e.g. *Sporolithon* *sp.* and *S.* *yoneshigueae*, both Sporolithaceae, which were grouped at similarity level of about 90% to the other grouping species (Fig. S2). Considering only the Abrolhos Bank, the ten species presented an 80% similarity in their Mg contents in the calcite lattice, with a high mean value of 20.9 (± 4.8) wt.% MgCO_3_ in comparison to warm temperate Brazilian region (e.g. Arvoredo Island).

Analysis of *Lithothamnion* *crispatum* along the Brazilian Shelf revealed that the mineralogy of samples from Abrolhos was distinct from those of the other four locations, Salvador, Espirito Santo, Trindade Island and Amazon River mouth (Fig. S3). Samples from the Abrolhos Bank presented higher amounts of high-Mg calcite (Fig. S4A). This pattern was also observed in the Mg-substitution in calcite in *L.* *crispatum*, which was 22.8 wt.%, higher in the Abrolhos sample (Fig. S4B) than the other four sites. On the contrary, mean aragonite content was lower in Abrolhos (Kruskal–Wallis, p = 0.02; Fig. S4C). The mean dolomite content was low in all samples (less than 3%), with the highest amount in the Espírito Santo (Kruskal–Wallis, p = 0.01; Fig. S4D).

The higher Mg substitution observed in *Lithothamnion* *crispatum* samples can be attibuted to the higher seawater temperature from the Abrolhos region, 27.95 ºC in average, compared with the average of the other regions (26.53 ºC). The relatioship between higher Mg substitution and higher temperature was also confirmed when comparing data from the regions with the highest sample size in the Brazilian Shelf, regardless of the coralline algae species (Fig. S5).

### Localization of different CaCO_3_ polymorphs within coralline algae skeleton

**Figure 1:**
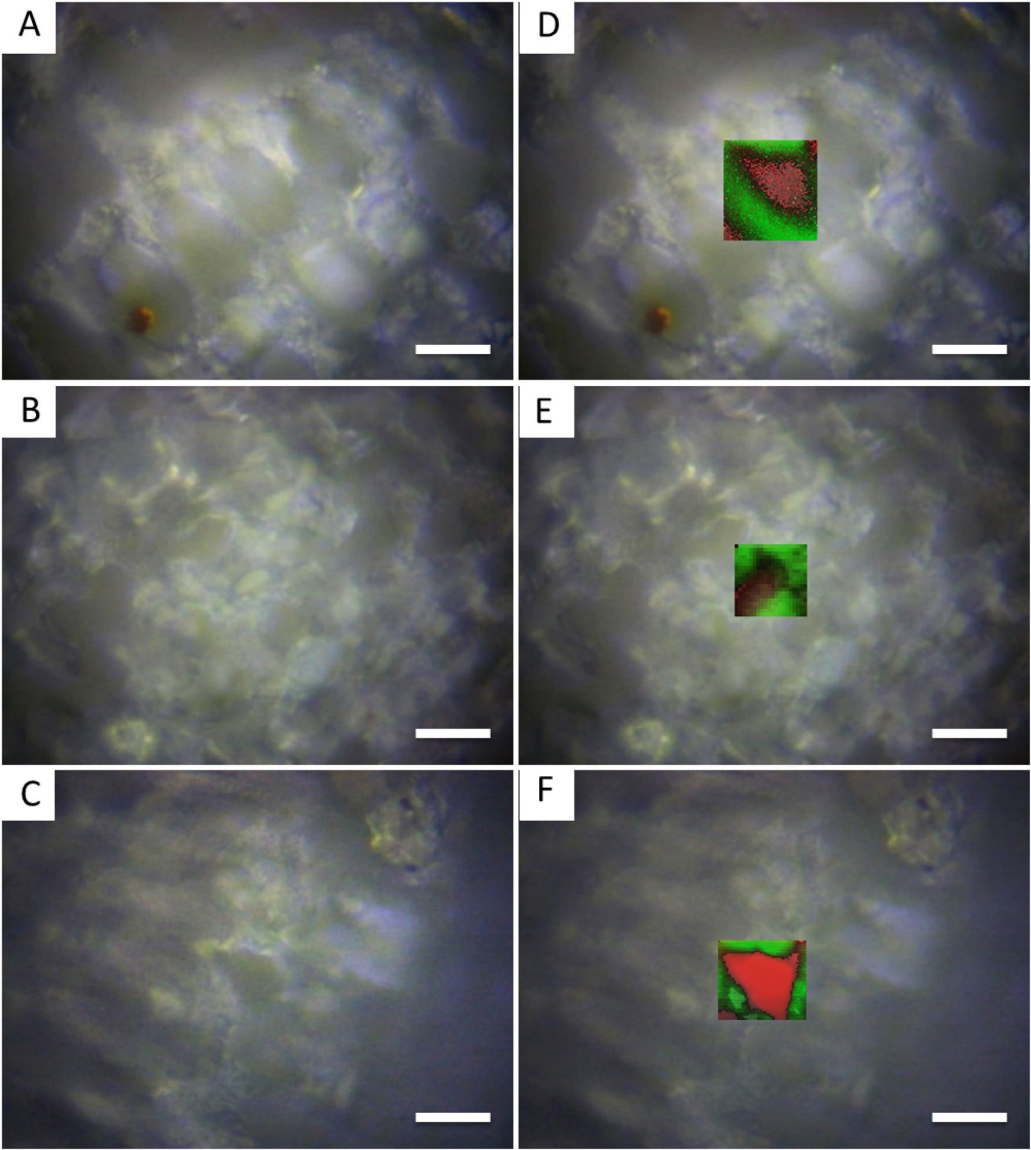
Bright field optical microscopy of the three samples from the three different families of the order Corallinales (Corallinaceae (**A)**; Hapalidiaceae (**B)**; and Sporolithaceae (**C)**). Bright field image overlaid Raman mapping indicates the same pattern from the three samples, with high-Mg calcite in the internal cell walls and aragonite as a “shell” close to the cell membrane (green: high-Mg calcite; red: aragonite). Scale bars: 20 µm.

Raman mapping was performed at polished coralline algae samples from *L.* *coralline* (Fig. 1A), *L.* *crispatum* (Fig. 1B) and *S.* *episporum* (Fig. 1C). The analysis revealed a similar pattern of the calcium carbonate polymorph distribution in cell walls of the three species (Fig. 1D–F). The regions corresponding to the outer cell walls were mainly composed of high-Mg calcite (green labeling). In contrast, the inner region of the cell wall (close to the cell limits) was composed of a “thin rim” of aragonite (red labeling).

### Comparison to mineralogical published data from other world regions

**Figure 2:**
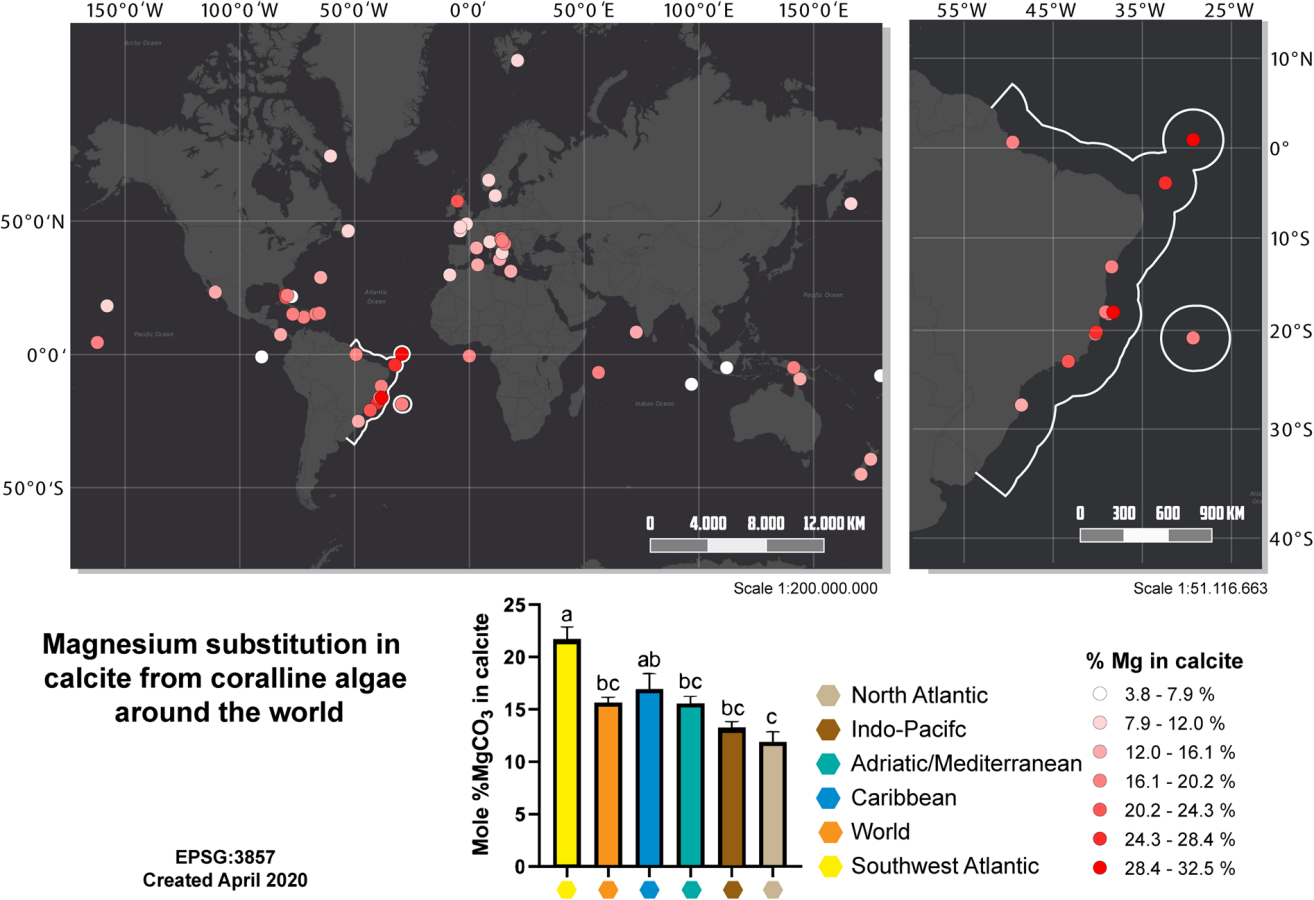
Map of world distribution of % Mg in calcite in coralline algae from four regions (North Atlantic Ocean, Indo-Pacific Ocean, Adriatic/Mediterrean Sea, Caribbean and Southwest Atlantic Ocean; upper left in the figure) ranging from 4 to 33% Mg in calcite; a detailed map of % Mg in calcite in coralline algae species analyzed in this study along the Brazilian shelf (upper righ in the figure); mean % Mg in calcite in coralline algae and standard deviation in several regions of world. Different letters are used to indicate statistically differences (p < 0.05) obtained through post-hoc Dunn’s test after the Kruskal–Wallis analysis (bellow in the figure). Both maps maps were obtained from ESRI database (https://services.arcgisonline.com/arcgis/rest/services/Canvas/World_Dark_Gray_Base/MapServer) and the coordinates from the mineralogy analysis were standardized using EPSG: 3857-WGS 84/Pseudo-Mercator. Maps were produced with QGIS v3.4 (https://www.qgis.org).

Quantitative mineralogical data were accessed using previous coralline algae mineralogical studies^11,21–25^ (Table S2). Previous mineralogical data of different seas/oceans, including the Caribbean Sea (e.g. Jamaica, Bahamas, Puerto Rico); the Mediterranean/Adriatic Sea, the Indo-Pacific Ocean (e.g. Great Barrier Reef, Papua New Guinea, New Zealand); the North Atlantic Ocean/Arctic Ocean (e.g. France north coast, Norway) were compared to each other (Fig. 2). From the 86 coralline species reported, the mineralogy of 14 species was determined for the first time in our study. The Southwestern Atlantic presented the highest Mg-substitution in calcite (46.3%; Kruskal–Wallis and Dunn’s test, p = 0.0008; Fig. 2) contrasting with the mean of 21.1 wt.% vs 14.42 wt.% in other species/regions. The Mg-substitution at the Southwest Atlantic Ocean was also higher than each region when compared to each other with exception of the Caribbean (Kruskal–Wallis and Dunn’s test, p = 0.8395). However, the Mg-substitution in the Caribbean was only higher than the North Atlantic (Kruskal–Wallis and Dunn’s test, p = 0.0314).

## Discussion

The data reported in this study expands upon the present knowledge concerning the mineralogy of coralline algae species worldwide, encompassing for the first time coralline algae species data from the Southwest Atlantic Ocean, where this group is the main frame-builders in coral reefs and the major inner component in rhodoliths^16,26^.

Mineralogical analysis revealed that coralline algae species of the Brazilian Shelf were mainly formed of high-Mg calcite. Six coralline algae species in this study had the same range of high-Mg calcite, between 80 and 100%, than the same species from different regions of the world: *Lithophyllum* *corallinae,* *Lithophyllum* *kaiseri* (as *Lithophyllum* *congestum*), *Lithophyllum* *stictaeforme,* *Lithothamnion* *crispatum*, *Melyvonnea* *erubecens* (as *Lithothamnion* *erubecens*) and *Sporolithon* *episporum* (Table S2). This result confirms that species from different families, such as Corallinaceae, Hapalidiaceae and Sporolithaceae have a CaCO_3_ skeleton formed mainly of high-Mg calcite.

In agreement with earlier studies, the average high-Mg calcite content in Corallinaceae was very similar to the results compiled by Smith et al*.*^11^ (96.7 wt.% and 96.2 wt.%, respectively). This pattern was also observed for Hapalidiaceae, which presented a mean value of 88.9 ± 3.6 wt.% in our study and 90.2 wt.%. However, Smith et al*.*^11^ registered a high-Mg calcite content of 98 wt.% for Sporolithaceae, while in our study this polymorph had a mean occurrence of 86.2 ± 6.5 wt.%. This percentage can be attributed mainly to the lower content of high-Mg calcite found in *Sporolithon* *yoneshigueae*, which is an endemic species of the Brazilian Shelf^27^.

The high similarity between the mineralogy (% high-Mg calcite, % aragonite and % dolomite) of the species belonging to three encrusting algae families, revealed by the cluster analysis, emphasizes the lack of CaCO_3_ disparities over skeleton mineralogy of coralline algae at family level. This aspect was also evidenced by several studies concerning coralline algae mineralogy^11,21–25^. This fact was confirmed in the cluster analysis between the mineralogy of the studied coralline species, in which samples from different families were grouped. Considering these findings, the mineralogical pattern exhibited by the crustose algae may not be driven by taxonomic classification, as was first proposed by Chave^28^. Therefore, the skeletal mineralogy from Brazilian coralline algae species can not be used as a taxonomic character, not even for higher taxonomic levels.

In this sense, the mineralogical analysis from *L.* *crispatum*, the most common rhodolith-forming species on the Brazilian Shelf^16^, revealed that samples from the Abrolhos Bank presented higher high-Mg calcite in their composition, and the highest % of Mg substitution in the calcite lattice than the species from the other four regions studied. One of the possible explanations is that the Abrolhos Bank has the highest seawater temperature compared to the other four sites, which influences CCA mineralogy. This result corroborates the hypothesis that coralline algae species do not have a strict control over Mg precipitation as stated by Stanley et al*.*^29^. In addition to seawater temperature, Mg/Ca ratio in seawater can also affect the incorporation of magnesium into coralline algae skeletons^11,29^.

In relation to other CaCO_3_ polymorphs, previous studies have registered some species with up to 20% aragonite^11,12^. Meanwhile, in this study, *S.* *yoneshigueae* presented CaCO_3_ skeletons formed of more than 30% of aragonite, which expands the range found in coralline algae for this polymorph. The high percentage of aragonite found in *S.* *yoneshigueae* could be related to the fact that this species presents larger overgrown calcified empty tetrasporangial compartments, in comparison with other Sporolithaceae species^27^, which could be filled with aragonite. This feature has mainly been described in the overgrown conceptacles of *Lithothamnion* sp.^30^ and in cell infills of *Porolithon* *onkodes*^31^. The presence of aragonite could be also attributed to the use of aragonite granules in the sediment to repair any damage in the alga-substrate attachment^32^.

Raman mapping showed the presence of high-Mg calcite in the bulk of the cell wall with little aragonite in its inner part, which seems to form an inner “shell”, closer to the cell membrane. To date, this is the first study that has utilized Raman maps to show the localization of aragonite in cell walls of coralline algae. The maps consisted of the cellular living layer from the coralline algae crust, right beneath the epithelial cells, which indicates that the mineralization of aragonite occurred in live cells and it was probably not a remineralization process.

Aragonite inside cell bodies was first seen by Nash et al*.*^12^ using Backscattered Scanning Electron Microscopy. They also reported the presence of dolomite or protodolomite, which were not observed herein by Raman spectroscopy, probably because of the low amount of this polymorph.

Previous studies considered that the inclusion of dolomite into carbonate skeletons is a microbial-mediated process after cell death upon the discovery of microbial-associated dolomite formation in anoxic marine^33^ and freshwater environments^34^. The presence of several calcium carbonate polymorphs found in coralline algae raises the question of whether all these polymorphs are in fact synthesized by the algae.

Indeed, the role of coralline algae in the different forms of calcium carbonate crystal precipitation is a crucial issue that should be addressed. Nowadays, studies calculate the production of CaCO_3_ by coralline algae based on CCA coverage^35^, without considering that not all CaCO_3_ produced in that structure is related to coralline algae biomineralization processes (e.g. secondary calcification processes such as infilling of the older skeleton and skeletal dissolution *vs* newly deposited carbonate). Therefore, it would be misleading to presume the net CaCO_3_ accretion of coralline algae structures without knowing the origin of the CaCO_3_ processes. This is also valid in relation to studies on the influences of atmospheric [CO_2_] rise on coralline algae, based on weight changes^36–38^ and its impacts on the mineralogy of the existing crust^21^.

Concerning Mg^2+^ substitution in the high-Mg calcite lattice, we found that Brazilian encrusting algae possess a higher Mg-substitution (46.3% more Mg^2+^ than the global average) in calcite than specimens collected worldwide_._ A possible explanation for the higher mean Mg^2+^ content might be related to the high seawater temperatures^39^, as this was also observed along the tropical Brazilian Continental Shelf. This can be exemplified by the high Mg^2+^ content found in fourteen species that occur in warmer waters of the Brazilian Shelf, where the mean surface seawater temperature (SST) ranged between 26.4 and 29.8 °C (from 2008 to 2016), between 17°S and 3°N. The lower Mg^2+^ amounts presented in *L.* *margaritae* and *L.* *attlanticum* could also be explained by the temperature, as these species were collected at the southernmost site (27°S) in the temperate zone, where the mean SST (from 2008 to 2016) varied between 22.5 and 25 °C (NOAA Comprehensive Large Array-Data Stewardship System-CLASS: SST50). A relationship between the Mg^2+^ content and temperature has already been proposed in previous works^39^ and is widely accepted. Nash and Adey^40^, when plotting the data collected using XRD, found a very strong correlation coefficient (R^2^ = 0.975) between mol% MgCO_3_ in coralline algae and temperature. Moreover, the Mg/Ca rate in coralline algae is used as a proxy archive^41^ and to generate multicentury-scale climate records from extratropical oceans^42^.

Although seawater temperature is loosely associated with latitude, the New Zealand species, for example, are subjected to lower temperatures (2012 annual maximum and minimum surface seawater temperatures: 21 and 18.7 °C, respectively), while Caribbean and Cocos Island algae grow at higher temperatures (2008–2016 annual maximum and minimum surface seawater temperatures: 29.5 and 23.4 °C, respectively) (NOAA Comprehensive Large Array-Data Stewardship System – CLASS: SST50). If we consider the differences in temperature (≅ 6 °C) and Mg^2+^ content difference (7.67 wt.%) between the sampling sites along the Brazilian Shelf, we can infer that there is an average increase of 1.27 wt.% of Mg^2+^ per °C. This value is in the range from 0.4 to 2 wt.% Mg per °C reported previously, both in experimentally and in situ studies^39^.

This relationship between Mg substitution and temperature is also critical in face of the temperature risen episodes that we are seeing all over the world^43^, including the Brazilian Shelf^44^. If coralline algae produces High Mg calcite with more Mg substitution in higher seawater temperatures, these thermal anomalies could force the production of a highly soluble polymorph, making coralline algae skeleton even more prone to dissolution.

It is well known that high-Mg calcite is the most soluble CaCO_3_ crystalline polymorph under acidified conditions and that this dissolution is more evident when Mg substitution in the calcite lattice is higher^45^. In our study 70% of the coralline algae species presented a Mg substitution in the range of 12 to 24% and the mean Mg substitution was 21.1%, which reinforces the susceptibility of Southwestern Atlantic coralline algae to future high [CO_2_] scenarios.

Even though previous experiments using synthetic calcium carbonate showed that the rise of seawater temperature increases Mg substitution, making high-Mg calcite more stable^46^ and other studies claiming that coralline algae with higher Mg substitution (more than 24% in average) presented less dissolution when exposed to high [CO_2_]^13^, Southwestern Ocean coralline algae are already living in a limit situation, where seawater can reach temperatures up to 28 ºC. Since we have a correlation between Mg substitution and temperature around 1.27% Mg per 1 ºC, it would take 2.4 to 6.2 ºC rise so the alga starts to produce a more stable calcite polymorph. Such a temperature rise could be lethal to these algae, also promoting a surface microbial shift that could be crucial to sucectional processes (e.g. settlement) involving other marine organisms, such as corals, which is critical for reef regeneration and recovery from climate-related mortality events^47^. The comparisons of results obtained through assays with synthetic calcium carbonate must be done with caution, because it should be take into account that the complex calcium carbonate biomineralization processes performed by marine organisms are highly dependent of a narrow range of environmental conditions.

In face of the dependency of these environmental conditions, the broad range of Mg content in temperate coralline algae^25^, a high inter species variability in the % Mg in this study (Abrolhos Bank; 14.5 to 28.8% Mg), as well as an anatomical difference in Mg content in coralline algae^40^, suggest that other environmental parameters (e.g. Mg/Ca in seawater, light, salinity, etc.) could also drive Mg substitution in coralline algae. Furthemore, coralline algae biological processes might exert some kind of control over Mg-calcite calcification which make them more resilient under rising CO_2_^39^.

Long-term projections of ocean acidification and the CaCO_3_ saturation state indicated that high-latitude seawater will be undersaturated with respect to high-Mg calcite in the second half of this century^45^. Early results with coralline algae *Sonderophycus* *capensis* and *Lithothamnion* *crispatum* in a subtropical mesocosm in Brazil showed that an increase in seawater pCO_2_ (1000 ppm) enabled both species to continue photosynthesizing but did cause carbonate dissolution^48^.

However, coralline algae from the North Atlantic Ocean, where the temperatures are lower, presented the lowest Mg substitution mean (11.91%), with some algae presenting only 8% of Mg substitution. This fact confers a more stable calcite skeleton to face ocean acidification then individuals from tropical environments. In addition, coralline algae from the Southwestern Atlantic Ocean are already living at temperatures that can be considered a limit for their survival. In fact, for cold water species, a subtle temperature increase could be beneficial in terms of their metabolism, photosynthesis and biomineralization.

By the year 2100, surface seawater in all climatic zones could be undersaturated or at metastable equilibrium, with a high-Mg calcite phase containing ≥ 12 mol% Mg^45^. This could be catastrophic to coralline algae from the Southwest Atlantic Ocean, which produce CaCO_3_ crystals with more than 20% of Mg substitution in average as shown by the present study and for all the carbonate structures (e.g. rhodolith beds, coralline reefs, etc.) that depends on these skeletons to maintain and grow.

It is worth to mention that coralline algae are present since the Mesozoic, in particular Sporolithaceans, which were already abundant in Cretaceous shallow waters^49^ and have already been submitted to bigger climate change events in the past, such as the Paleocene-Eocene Thermal Maximum (PETM), in which the deep-water temperature increased ∼5 ºC and a massive carbon cycle change took place with a large amount of CO_2_ absorbed by the oceans^50^. One of the possible explanations for the survival of coralline algae is that their biomineralogical control is limited to polymorph specification and would be ineffectual in the regulation of skeletal Mg incorporation^51^. In this sense, in past geological eras, such as the Cretaceous and Paleogene, the Mg/Ca ratio of the oceans favors the precitation of low Mg calcite^29,52^, which are more stable to dissolution. In a parallel to present day, other fundamental aspect we should take into account is the speed of progression of these changes. Actually, we know that the fast evolution of temperature and acidification present scenarios may result in significant impact on marine biodiversity and in marine calcium carbonate cycle players, as reef organisms and CCA.

Carvalho et al.^53^ proposed that there would be a suitable area for rhodolith occurrence around 230,000 km^2^, providing a new magnitude to Brazilian Continental Shelf relevance as a major world biofactory of carbonate. In fact, this work confirms the estimation from previous studies, which indicated that this area would correspond to a 2 × 10^11^ tons of carbonate deposit of the Brazilian coast^53^. Among the most critical regions in the Brazilian coast, the Abrolhos Bank encompasses the largest continuous latitudinal rhodolith beds registered to date^6^, which is responsible for the production of approximately 0.025 Gt^−1^ year^−1^ of calcium carbonate, similar to those values reported for major tropical reef environments^54,55^. Another recently described important reef area on the Brazilian Shelf is an extensive carbonate system (≅ 9500 km^2^) off the Amazon River mouth^56^, which is composed of mesophotic carbonate reefs and rhodolith beds. These huge carbonate reservoirs and biodiversity hotspots may undergo a major decline if global ocean acidification and temperature rise take place in the near future.

## Conclusions

This work corroborates with previous studies, showing that coralline algae possess minimum control over its biomineralization process, which can be critical to its resilience when facing the eminent impact of changes in seawater physico-chemical properties.

The description of the CCA mineralogy and cell wall structural modifications under warming and acidification (formation of thinner tissue crusts, lower ratios of calcitic/organic matrix, and cell wall narrowing) as well as the high Mg-calcite calcification process is essential for indicating the more susceptible areas of the world to ocean acidification.

The mean Mg-substitution of encrusting coralline algae from the Brazilian Shelf was 46.3% higher than global average. Because of the higher mean Mg-substitution in calcite compared with worldwide coralline algae species, these algae from Southwest Atlantic Ocean would be highly susceptible to dissolution which will be caused by the expected near-future ocean acidification levels and negatively will compromise CaCO_3_ net production across the Brazilian Shelf. Considering the mean high-Mg substitution (21.1%) found in coralline algae in this region, the Southwest Atlantic Ocean can be considered as the most threatened region in comparison to other tropical and temperate areas worldwide.

## Methods

### Herbarium and live samples analysed

Thirty coralline algae specimens were selected from RB and FLOR herbaria collections (Voucher specimens in Table S1), as well as previous sampling performed at different sites along the Brazilian Shelf between 2008 and 2019. Sample collection localities ranged from 0.44°S, at the Amazon River mouth, to 27.29°S in the Arvoredo Island. Specimens were identified by morpho-anatomical characters observed in thin sections prepared following previous protocols^57^.

### Mineralogical analysis

Superficial tissue was collected using a razor blade without digging into, scraping or scoring the skeleton. Three fragments (≅ 1 cm^2^) of each specimen were washed and scraped free of debris or encrusting organisms, and further separated and individualized in 1.5 ml microtubes. Samples were not bleached. Each sample was ground to a fine powder and sieved through 25 μm mesh previously to X-Ray Diffraction (XRD) for calcium carbonate mineralogy.

The X-ray data were collected using a PANalytical X’Pert Pro Multipurpose Powder Diffractometer (Bragg–Brentano geometry, CuKα radiation, generator settings: 40 mA and 40 kV). An angular range of 5° to 90° 2θ was measured with a step size of 0.02° and 1°/min speed. Samples were prepared in triplicate, and each replicate sample was measured. Phase identification was performed with Panalytical X’Pert Pro V3 software. A “search and match” algorithm was used with organic and inorganic databases to identify the crystals by their diffraction peaks. Automated identifications were confirmed or refuted/amended using a technical analysis of the diffraction peaks. Phase quantification and lattice parameter determination were performed (N = 5 interactions) with the Rietveld refinement software MAUD, which was also used to determine the percentage of Mg-substitution following Titschack et al.^58^, using *c*, the *c/a* ratio, cell volume and (104) peak dislocation as refinement parameters.

### Characterization of coralline algae families by XRD and Raman microspectroscopy

**Figure 3:**
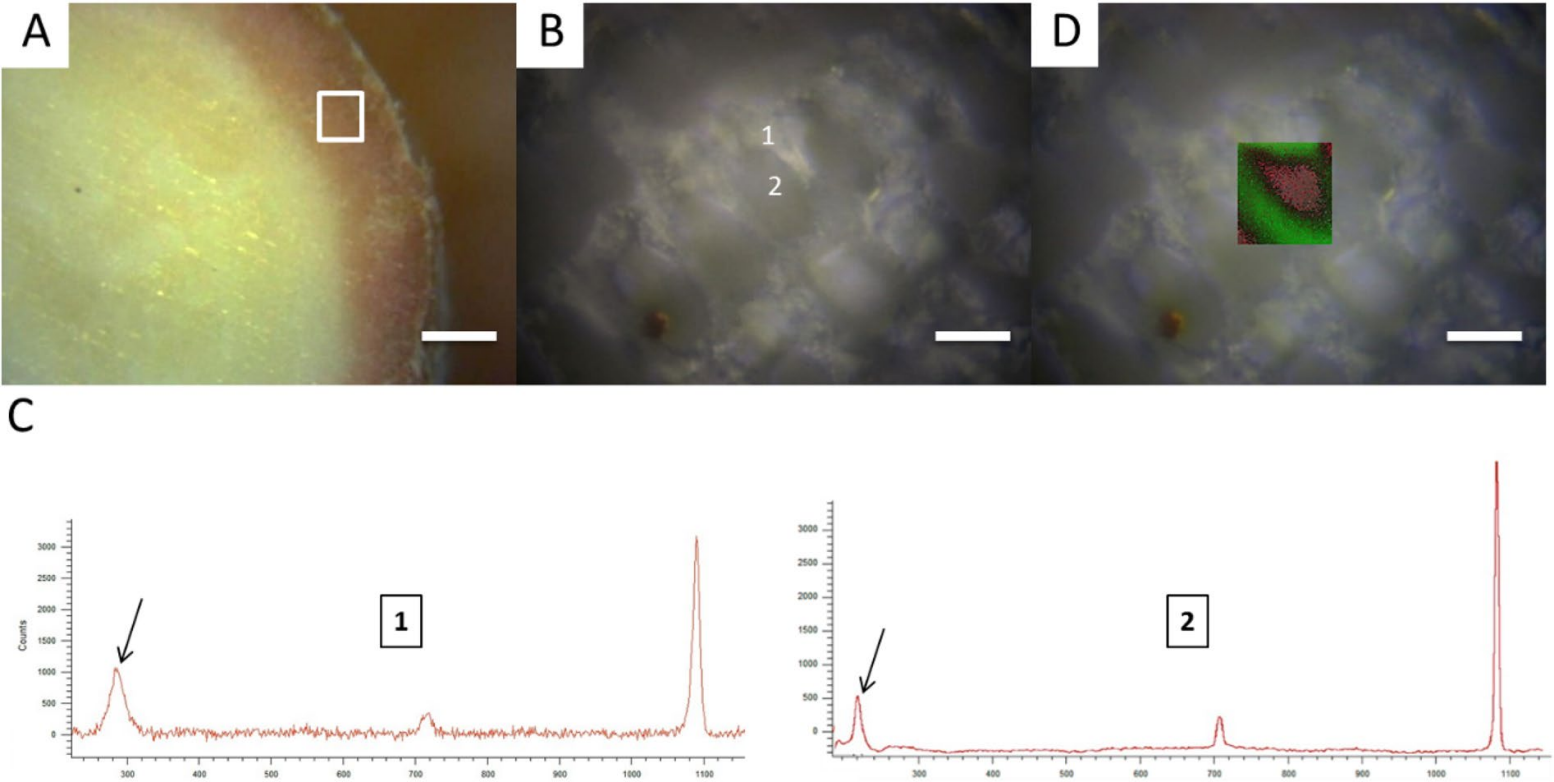
Low magnification view of coralline algae thallus with the selected area for Raman analysis marked (white square) (**A**; scale bar: 200 µm). Optical microscopy image of coralline algae cells with numbers 1 and 2 representing the punctual Raman analysis of the cell (**B**; scale bar: 20 µm) and their respective Raman spectra with a black arrow indicating the peaks that were used to differentiate high-Mg calcite (**C**; black arrow indicates the 281 cm^−1^ calcite peak) from aragonite (2; black arrow indicates the 208 cm^−1^ aragonite peak). Optical microscopy image overlaid by Raman mapping shows the prevalence of high-Mg calcite (green) in the internal cell wall and aragonite (red) as a “shell” in the inner part of the cell (**D**).

Small fragments (n = 9, 3 per family) of coralline algae species from each of the three families, Corallinaceae (*Lithophyllum* *corallinae*); Hapalidiaceae (*Melyvonnea* *erubecens*, as *Mesophyllum* *erubescens*); Sporolithaceae (*Sporolithon* *episporum*) were analyzed by Raman Spectroscopy in order to localize the main calcium carbonate polymorphs that form the skeleton of living coralline algae. *Lithophyllum* *corallinae* (Corallinaceae), *L.* *crispatum* (Hapalidiaceae) and *Sporolithon* *episporum* (Sporolithaceae) represent widely distributed species along the Brazilian shelf and are important reef and rhodolith builders^16^. The samples were washed of debris and epiphytes, polished and sonicated before analysis (Fig. 3A). Spectroscopic data were obtained using a Renishaw Invia Raman system with macro- and confocal micro-Raman capabilities. Owing to the confocal capability, the use of a 100× objective (having an extended working distance of 3.4 mm, a numerical aperture of 0.8, and not requiring immersion oil) provided a horizontal resolution of 1 μm and a vertical resolution of 1–3 μm. Samples were centered in the path of the laser beam projected through a Leica DM IRB microscope (Leica, Germany; Fig. 3B). The typical laser power was 3 mW, an instrumental configuration below the threshold causing radiation damage to live specimens studied here. The equipment was coupled with a 532 nm Ar laser source and a 785 nm laser diode source. As coralline algae cell components auto fluorescence blues Raman analyses when using the 532 nm laser source, we used the 785 nm laser source instead. Beam centering and Raman spectra calibration were performed daily on a Si chip displaying a characteristic Si Raman shift at 520.4 cm^−1^. Extended mode analysis in the range of 200–1400 cm^−1^ was used to acquire reference spectra (Fig. 3C). Calcite regions were identified by the 284 cm^−1^ peak (Fig. 3C: 1, black arrow) and aragonite by the 208 cm^−1^ peak (Fig. 3C: 2, black arrow). Twenty individual accumulations of 5 s integration time were performed in order to obtain each reference spectrum. Dynamic line-scanning high resolution Raman mapping (Renishaw Streamline-HR) was performed in the range 200–1400 cm^−1^ by scanning the sample over selected areas (20 s pt^−1^) by using a motorized PRIORTM stage allowing XYZ displacements with a sub-micrometric step precision (Fig. 3D). Laser intensity was set at 3 mW. Light was dispersed by a holographic grating with 1200 grooves per millimeter and the signal was analyzed with a RENCAM charge-coupled device detector. Compositional maps representing the intensity distributions of characteristic peaks were determined using the software Wire 3.2 (Renishaw) based on component analysis, comparing reference spectra with each spectrum obtained. Spectra collected on the three coralline algae species were identified by comparison with pure aragonite and calcite mineral standards from the mineral database provided by Renishaw.

### Clustering of algae families and species based on CaCO_3_ mineralogy

The similarity between coralline algae at family/species taxonomic level and their mineralogy was assessed by cluster analysis. To perform this analysis, data regarding the proportion of high-Mg calcite, aragonite, dolomite and the amount of Mg-substitution at the calcite lattice were used. A Bray–Curtis resemblance matrix was created and a hierarchical cluster analysis (CLUSTER) was performed on the resemblance matrix with a SIMPROF test to determine sample group structure. The analyses were executed using PRIMER 6.0 software^59^.

In order to identify intraspecific mineralogical association driven by different collection sites, individuals (n = 3) of the same species (*Lithothamnion* *crispatum*) from five different collection sites (Amazonia, Abrolhos, Salvador, Vitoria and Trindade Island) were analyzed by powder XRD. The results were also determined using a hierarchical cluster analysis and SIMPROF test.

### Compilation of mineralogical data from studied crustose coralline algae (CCA)

A comprehensive literature review was performed using the web engines, *Web* *of* *Science* (Thomson Reuters, New York, NY) and *Google* *Scholar* (Alphabet Inc., Mountain View, CA), looking for articles presenting mineralogical information of CCA, mainly from those species also studied in this work, between 1920 and 2018 and using the following topics: “species name” and “mineralogy” or “species name” and “diffraction” or “species name” and “calcite” or “species name” and “Mg” or “coralline algae” and “mineralogy” or “coralline algae” and “diffraction” or “coralline algae” and “calcite” or “coralline algae” and “Mg”. A table and a global map were prepared with Mg-substitution in calcite data obtained in this study and previous papers. For mapping the mineralogy, a global map was produced with QGIS 3.4 software using World Dark Gray Base layer designed by ESRI (https://services.arcgisonline.com/arcgis/rest/services/Canvas/World_Dark_Gray_Base/MapServer). Coordinates of the world mineralogy analysis were standardized withEPSG: 3857-WGS 84/Pseudo-Mercator.. The spatial resolution of the map was a grid defined by cells of 2' latitude and longitude. The % Mg substitution in calcite was divided into ranges of 4% from 4 to 35% wt. and each range was assigned to each grid cell according to the geographic coordinate of the sampling site using IOS map app. Mineralogy data from the world and each region (Southwest Atlantic, North Atlantic, Caribbean, Mediterranean/Adriatic and Indo-Pacific Ocean) were compared using (GraphPad Prism version 8.0.0 for Windows).

## Supplementary Information

Supplementary Information 1.

Supplementary Information 2.

## Data Availability

The datasets generated during and/or analysed during the current study are available from the corresponding author on reasonable request.
