# Supplementary material for: Global assessment of coralline algae mineralogy points to high vulnerability of Southwestern Atlantic reefs and rhodolith beds to ocean acidification
Source: Sci Rep. 2022 Jun 10;12:9589. doi: 10.1038/s41598-022-13731-y (PMC9187768; doi:10.1038/s41598-022-13731-y)
Supplement: Supplementary file 2 — Supplementary Information 2. [file 41598_2022_13731_MOESM2_ESM.docx]

Supporting Information: Rodrigo Tomazetto de Carvalho, Gustavo Miranda Rocha, Cláudia Santiago Karez, Ricardo da Gama Bahia, Renato Crespo Pereira, Alex Cardoso Bastos, Leonardo Tavares Salgado

**Global assessment of coralline algae mineralogy points to high vulnerability of Southwestern Atlantic reefs and rhodolith beds to ocean acidification**

Table S1: List of all the specimens collected for this study, their respective collection sites and Herbarium Voucher.

| Specimen | Collection Site | Voucher | | |
| --- | --- | --- | --- | --- |
| *Amphiroa anastomosans* | Espírito Santo | N.I.* | | |
|  | (19º54’00”S, 40º05’53”W) |  |  |  |
| *Amphiroa brasiliana* | Espírito Santo | N.I.* | | |
|  | (19º55’32”S, 40º06’13”W) |  |  |  |
| *Amphiroa fragilissima* | Espírito Santo | N.I.* | | |
|  | (19º55’93”S, 40º07’16”W) |  |  |  |
| *Amphiroa rígida* | Espírito Santo | N.I.* | | |
|  | (19º58’32”S, 40º08’18”W) |  |  |  |
| *Corallina panizzoi* | Espírito Santo | N.I.* | | |
|  | (19º59’99”S, 40º08’82”W) |  |  |  |
| *Harveylithon rupestre* (as *Hydrolithon rupestre*) | Saint Peter & Saint Paul Archipelago | RB 553503 | | |
|  | (0º91’73”N, 29º34’57”W) |  |  |  |
| *Jania adhaerens* | Espírito Santo | N.I.* | | |
|  | (20º02’05”S, 40º09’32”W) |  |  |  |
| *Jania cubensis* | Espírito Santo | N.I.* | | |
|  | (20º02’14”S, 40º10’22”W) |  |  |  |
| *Jania subulata* | Espírito Santo | N.I.* | | |
|  | (20º04’40”S, 40º10’49”W) |  |  |  |
| *Lithophyllum attlanticum* | Arvoredo Island | FLOR 14568 | | |
|  | (27º16’32”S, 48º22’74”W) |  |  |  |
| *Lithophyllum corallinae* | Rio de Janeiro | N.I.* | | |
|  | (22º95’31”S, 43º16’12”W) |  |  |  |
| *Lithophyllum kaiseri* (as *Lithophyllum* *congestum*) | Abrolhos | RB 382794 | | |
|  | (17º57’50”S, 38º42’02”W) |  |  |  |
| *Lithophyllum margaritae* | Arvoredo Island | FLOR 14570 | | |
|  | (27º16’32”S, 48º22’74”W) |  |  |  |
| *Lithophyllum stictaeforme* | Abrolhos | RB 525142 | | |
|  | (17º57’45”S, 38º41’43”W) |  |  |  |
| *Lithothamnion crispatum* | Vitória | RB 623157 | | |
|  | (20º31’61”S, 40º27’11”W) |  |  |  |
| *Lithothamnion crispatum* | Salvador | RB 563698 | | |
|  | (12º58’01”S, 38º22’13”W) |  |  |  |
| *Lithothamnion crispatum* | Amazon River Mouth | N.I.* | | |
|  | (3º31’07N, 49º95’15”W) |  |  |  |
| *Lithothamnion crispatum* | Abrolhos | RB 525146 | | |
|  | (17º57’45”S, 38º41’43”) |  |  |  |
| *Lithothamnion crispatum* | Trindade Island | RB 563699 | | |
|  | (20º30’13”S, 29º21’19”W) |  |  |  |
| *Lithothamnion muelleri* | Abrolhos | RB 564946 | | |
|  | (17º57’45”S, 38º40’18”W) |  |  |  |
| *Melyvonnea erubescens* (as *Mesophyllum erubescens*) | Abrolhos | RB 623166 | | |
|  | (17º57’45”S, 38º41’43”W) |  |  |  |
| *Melyvonnea erubescens* (as *Mesophyllum erubescens*) | Aracruz | RB 537866 | |  |
|  | (3º87’19”S, 32º41’46”W) |  |  |  |
| *Neogoniolithon* sp. | Abrolhos | RB 581400 | |  |
|  | (17º57’45”S, 38º41’49”W) |  |  |  |
| *Pneophyllum conicum* | Abrolhos | RB 621929 | |  |
|  | (17º57’45”S, 38º40’18”W) |  |  |  |
| *Porolithon onkodes* | Abrolhos | RB 623155 | |  |
|  | (17º47’00”S, 39º03’05”) |  |  |  |
| *Porolithon onkodes* | Salvador | RB 623151 | |  |
|  | (12º95’70”S, 38º35’92”W) |  |  |  |
| *Sporolithon episporum* | Abrolhos | RB 525149 | |  |
|  | (17º47’00”S, 39º03’05”W) |  |  |  |
| *Sporolithon ptychoides* | Abrolhos | RB 621750 | |  |
|  | (3º87’19”S, 32º41’46”W) |  |  |  |
| *Sporolithon* sp. | Campos | RB 614603 | |  |
|  | (21º88’40”S, 40º94’43”W) |  |  |  |
| *Sporolithon yoneshigueae* | Abrolhos | RB 621753 | |  |
|  | (17º48’35”S, 38º11’03”W) |  |  |  |

*NI= Not included yet.

Table S2: Mineralogy of CCA in this study (bold letters) collected from the Southwest Atlantic Ocean (Brazil) and in other studies from four main regions: The Caribbean Sea; the Adriatic/Mediterranean Sea; the Indo-Pacific Ocean; and the North Atlantic Ocean, including the Arctic Ocean.

| **Family** | **Genus** | **Species** | **Location** | **Region** | **Wt% Calcite** | **Wt% MgCO_3_** | **Source** |
| --- | --- | --- | --- | --- | --- | --- | --- |
| Corallinaceae | *Neogoniolithon* | *acropetum* | Culebra Island, Puerto Rico | Caribbean | 100 | 19.24 | Clarke & Wheeler, 1922 (as *Goniolithon acropetum*) |
|  |  | *brassica-florida* | New Zeland | Pacific Ocean | 97 to 99 | 13.1 | Smith *et al*., 2011 |
|  |  | ***sp.*** | **Abrolhos Bank, Brazil** | **Southwest Atlantic Ocean** | **99.3** | **25.7** | **This study** |
|  |  | *fratescens* | Cocos-Keeling Islands, Indian Ocean | Indian Ocean | 100 | 13.8 | Clarke & Wheeler, 1922 (as *Goniolithon frutescens*) |
|  |  | *orthoblastum* | Murray Island, Torres Straits, Australia | Pacific Ocean | 100 | 13.7 | Clarke & Wheeler, 1922 (as *Goniolithon orthoblastum*) |
|  |  | *strictum* | Bemini Harbour, Bahamas | Caribbean | 100 | 24 | Clarke & Wheeler, 1922 (as *Goniolithon strictum*) |
|  |  |  | Soldiers Key, Florida | Caribbean | 100 | 25.2 | Clarke & Wheeler, 1922 (as *Goniolithon strictum*) |
|  |  |  | Bemini Harbour, Bahamas | Caribbean | 100 | 23.3 | Clarke & Wheeler, 1922 (as *Goniolithon strictum*) |
|  | ***Harveylithon*** | ***rupestre*** | **Saint Peter & Saint Paul Islands, Brazil** | **Southwest Atlantic Ocean** | **87.5** | **32.5** | **This study (*)** |
|  | *Hydrolithon* | *craspedium* | Palmyra Island, Pacific | Pacific Ocean | 100 | 19.6 | Clarke & Wheeler, 1922 (as *Lithophyllum craspedium*) |
|  | ***Lithophyllum*** | ***attlanticum*** | **Arvoredo Island, Brazil** | **Southwest Atlantic Ocean** | **95.7** | **16.4** | **This study (*)** |
|  |  | *antillarum* | Culebra Island, Puerto Rico | Caribbean | 100 | 16.4 | Clarke & Wheeler, 1922 |
|  |  | *byssoides* | Losini, middle Adriatic | Adriatic/Mediterrenean | 98 | 16 to 20 | Medakovic *et al.*, 1995 (as *Lithophyllum lichenoides*) |
|  |  |  | Kornati, middle Adriatic | Adriatic/Mediterrenean | 98 | 16 to 20 | Medakovic *et al*., 1995 (as *Lithophyllum lichenoides*) |
|  |  |  | Ustica Island, Italy | Adriatic/Mediterrenean | 98,5 | 14 | Mannino, 2003 |
|  |  | *carpophylli* | New Zeland | Pacific Ocean | 88 to 100 | 12 to 16 | Smith *et al*., 2012 |
|  |  | ***corallinae*** | New Zeland | Pacific Ocean | 80 to 100 | 12 to 16 | Smith *et al.,* 2012 |
|  |  |  | **Rio de Janeiro, Brazil** | **Southwest Atlantic Ocean** | **97.9** | **25.2** | **This study** |
|  |  | *incrustans* | Gatteville, Manche, France | North Atlantic Ocean | 100 | 10.8 | Clarke & Wheeler, 1922 (as *Lithothamnion incrustans*) |
|  |  |  | Mazagan, Morocco | Adriatic/Mediterrenean | 100 | 9.8 | Clarke & Wheeler, 1922 (as *Lithothamnion incrustans*) |
|  |  |  | Banyuls, Mediterranean, France | Adriatic/Mediterrenean | 100 | 14.4 | Clarke & Wheeler, 1922 (as *Lithothamnion incrustans*) |
|  |  | *intermedium* | Fort Clarence, near Kingston, Jamaica | Caribbean | 100 | 16.6 | Clarke & Wheeler, 1922 |
|  |  | ***kaiseri*** | Salinas Bay, near Guanica, Puerto Rico | Caribbean | 100 | 19 | Clarke & Wheeler, 1922 (as *Lithophyllum daedaleum*) |
|  |  |  | **Abrolhos, Brazil** | **Southwest Atlantic Ocean** | **98.6** | **15.8** | **This study** |
|  |  | ***margaritae*** | **Arvoredo Island, Brazil** | **Southwest Atlantic Ocean** | **98.4** | **13.8** | **This study (*)** |
|  |  | *oncodes* | Coetivy Island, Indian Ocean | Indian Ocean | 100 | 18.2 | Clarke & Wheeler, 1922 |
|  |  | *pachydermum* | Dollar Harbour, South Cat Cay, Bahamas | Caribbean | 100 | 18.5 | Clarke & Wheeler, 1922 |
|  |  | *pallescens* | Bay of La Paz, Gulf of California | Pacific Ocean | 100 | 15.5 | Clarke & Wheeler, 1922 |
|  |  | *polymorphum* | Kattegat | North Atlantic Ocean | 100 | 9.1 | Clarke & Wheeler, 1922 (as *Lithothamnium polymorphum*) |
|  |  | *postulatum* | New Zeland | Pacific Ocean | 95 to 100 | 12 to 15 | Smith *et al*., 2011 |
|  |  | *racemus* | Istria, North Adriatic | Adriatic/Mediterrenean | 90 | 16 to 20 | Medakovic *et al*., 1995 |
|  |  |  | Cres Island, middle Adriatic | Adriatic/Mediterrenean | 100 | 16 to 20 | Medakovic *et al.*, 1995 |
|  |  |  | Kornati, middle Adriatic | Adriatic/Mediterrenean | 100 | 16 to 20 | Medakovic *et al.*, 1995 |
|  |  |  | Naples, Italy | Adriatic/Mediterrenean | 100 | 11.3 | Clarke & Wheeler, 1922 (as *Lithothamnium racemus*) |
|  |  |  | Bahamas | Caribbean | 100 | 5.4 | Clarke & Wheeler, 1922 |
|  |  | *riosmenae* | New Zeland | Pacific Ocean | 98 | 12.7 | Smith *et al*., 2012 |
|  |  | ***stictaeforme*** | New Zeland | Pacific Ocean | 90 to 100 | 13 to 15 | Smith *et al*., 2012 |
|  |  |  | **Abrolhos Bank, Brazil** | **Southwest Atlantic Ocean** | **96.8** | **17.8** | **This study** |
|  |  | sp. | Mediterranean | Adriatic/Mediterrenean | 100 | 12.8 | Clarke & Wheeler, 1922 |
|  |  |  | New Zeland | Pacific Ocean | 90 to 100 | 13 to 16 | Smith *et al*., 2012 |
|  |  | *pygmaeum* | New Guinea | Pacific Ocean | 100 | 20 | Clarke & Wheeler, 1922 (as *Lithophyllum tamiense*) |
|  | *Mastophora* | *pacifica* | New Zeland | Pacific Ocean | 100 | 13.5 | Smith *et al.*, 2012 |
|  | ***Pneophyllum*** | ***conicum*** | **Abrolhos Bank, Brazil** | **Southwest Atlantic Ocean** | **90.4** | **25.7** | **This study (*)** |
|  | ***Porolithon*** | ***onkodes*** | Coetivy I., Indian Ocean | Indian Ocean | 100 | 18.2 | Clarke & Wheeler, 1922 (as *Lithophyllum onkodes*) |
|  |  |  | Dollar Harbour, South Cat Cay, Bahamas | Caribbean | 100 | 18.5 | Clarke & Wheeler, 1922 (as *Lithophyllum pachydermum*) |
|  |  |  | Culebra Island, Puerto Rico | Caribbean | 100 | 16.4 | Clarke & Wheeler, 1922 (as *Lithophyllum antillarum*) |
|  |  |  | **Abrolhos Bank, Brazil** | **Southwest Atlantic Ocean** | **92.5** | **24.2** | **This study** |
|  | *Pseudolithophyllum* | *expansum* | Istria, North Adriatic | Adriatic/Mediterrenean | 94.6 | 16 to 20 | Medakovic *et al*., 1995 |
|  |  |  | Ist Island, middle Adriatic | Adriatic/Mediterrenean | 100 | 16 to 20 | Medakovic *et al*., 1995 |
|  |  |  | Kornati, middle Adriatic | Adriatic/Mediterrenean | 15 | 16 to 20 | Medakovic *et al*., 1995 |
|  | *Tenarea* | *tortuosa* | Genoa, Italy | Adriatic/Mediterrenean | 100 | 9.3 | Clarke & Wheeler, 1922 (as *Lithothamnion tortuosum*) |
|  |  |  | Naples, Italy | Adriatic/Mediterrenean | 100 | 11.6 | Clarke & Wheeler, 1922 |
|  |  |  | Kornati, middle Adriatic | Adriatic/Mediterrenean | 100 | 16 to 20 | Medakovic *et al*., 1995 (as *Tenarea undulosa*) |
|  | *Spongites* | *fruticulosus* | Naples, Italy | Adriatic/Mediterrenean | 100 | 8.1 | Clarke & Wheeler, 1922 (as *Lithothamnion ramulosum*) |
| Hapalidiaceae | ***Lithothamnion*** | ***crispatum*** | New Zeland | Pacific Ocean | 86 | 10.8 | Smith *et al*., 2011 |
|  |  |  | **Abrolhos Bank, Brazil** | **Southwest Atlantic Ocean** | **88.8** | **22.8** | **This study** |
|  |  |  | **Amazon river mouth, Brazil** | **Southwest Atlantic Ocean** | **79.5** | **19.2** | **This study** |
|  |  |  | **Salvador, Bahia, Brazil** | **Southwest Atlantic Ocean** | **79.9** | **16.4** | **This study** |
|  |  |  | **Trindade Island, Brazil** | **Southwest Atlantic Ocean** | **80.3** | **16.4** | **This study** |
|  |  |  | **Vitória, Espírito Santo, Brazil** | **Southwest Atlantic Ocean** | **76.7** | **16.2** | **This study** |
|  |  | *fornicatum* | Norway | North Atlantic Ocean | 100 | 9.3 | Clarke & Wheeler, 1922 |
|  |  | *glaciale* | Isle of Arran, Loch Sween | North Atlantic Ocean | 100 | 12.9 to 24.6 | Kamenos *et al*., 2008 |
|  |  |  | Conception Bay, Newfoundland | North Atlantic Ocean | 100 | 10.9 | Clarke & Wheeler, 1922 |
|  |  |  | Arctic Ocean | Artic Ocean | 100 | 13.2 | Clarke & Wheeler, 1922 |
|  |  | *kaiseri* | Cocos-Keeling Islands, Indian Ocean | Indian Ocean | 100 | 15.3 | Clarke & Wheeler, 1922 (as *Lithophyllum kaiseri*) |
|  |  | ***muelleri*** | **Abrolhos Bank, Brazil** | **Southwest Atlantic Ocean** | **89.7** | **14.5** | **This study (*)** |
|  |  | *nodosum* | no locality stated | - | 100 | 6.1 | Clarke & Wheeler, 1922 |
|  |  | *Tophiforme* | Arctic Ocean | North Atlantic Ocean | 100 | 9.6 | Clarke & Wheeler, 1922 (as *Lithothamnium soriferum*) |
|  |  | sp. | Bay of Naples | Adriatic/Mediterrenean | 100 | 4.2 | Clarke & Wheeler, 1922 |
|  |  |  | Java Sea | Pacific Ocean | 100 | 3.8 | Clarke & Wheeler, 1922 |
|  |  |  | Galapagos Islands | Pacific Ocean | 100 | 6.5 | Clarke & Wheeler, 1922 |
|  |  |  | Spitsbergen | Artic Ocean | 100 | 8.7 | Clarke & Wheeler, 1922 |
|  |  |  | Honolulu | Caribbean | 100 | 9.4 | Clarke & Wheeler, 1922 |
|  |  |  | Bering Islands | Pacific Ocean | 100 | 9.9 | Clarke & Wheeler, 1922 |
|  |  |  | Bermuda | Caribbean | 100 | 12.4 | Clarke & Wheeler, 1922 |
|  | *Melobesia* | sp. | coast of Algeria | Adriatic/Mediterrenean | 100 | 14.4 | Clarke & Wheeler, 1922 |
|  | *Mesophyllum* | *expansum* | Istria, North Adriatic | Adriatic/Mediterrenean | 94,6 | 16 to 20 | Medakovic *et al*., 1995 (as *Pseudolithophyllum expansum*) |
|  |  |  | Ist Island, middle Adriatic | Adriatic/Mediterrenean | 100 | 16 to 20 | Medakovic *et al.*, 1995 (as *Pseudolithophyllum expansum*) |
|  |  |  | Kornati, middle Adriatic | Adriatic/Mediterrenean | 15 | 16 to 20 | Medakovic *et al*., 1995 (as *Pseudolithophyllum expansum*) |
|  |  | *engelhartii* | New Zeland | Pacific Ocean | 96 to 100 | 12 to 15 | Smith *et al.,* 2011 |
|  |  | *erubecens* | New Zeland | Pacific Ocean | 92 to 100 | 11 to 13 | Smith *et al.,* 2011 |
|  |  | *macroblastum* | New Zeland | Pacific Ocean | 73 to 100 | 11 to 15 | Smith *et al.,* 2011 |
|  |  | *philippii* var *funafutiensis* | Funafuti Atoll | Pacific Ocean | 100 | 5.9 | Clarke & Wheeler, 1922 (as *Lithothamnium philipi var. funafutiensis*) |
|  |  | *printzianum* | New Zeland | Pacific Ocean | 73 to 100 | 11 to 16 | Smith *et al.*, 2012 |
|  |  | sp. | New Zeland | Pacific Ocean | 91.8 | 11.9 | Smith *et al*., 2012 |
|  |  |  | New Zeland | Pacific Ocean | 91 to 100 | 11 to 13 | Smith *et al*., 2012 |
|  | ***Melyvonnea*** | *erubecens* | New Zeland | Pacific Ocean | 92 to 100 | 11 to 13 | Smith *et al*., 2012 |
|  |  |  | Haingsisi, near Timor | Pacific Ocean | 100 | 16.9 | Clarke & Wheeler, 1922 (as *Lithothamnion erubecens*) |
|  |  |  | **Abrolhos Bank, Brazil** | **Southwest Atlantic Ocean** | **95.2** | **17.8** | **This study** |
|  |  |  | **Aracruz, Espírito Santo, Brazil** | **Southwest Atlantic Ocean** | **93.7** | **25.5** | **This study** |
|  | *Phymatolithon* | *calcareum* | Isle of Arran, Loch Sween | North Atlantic Ocean | 100 | 14.7 to 23.8 | Kamenos *et al.,* 2008 |
|  |  |  | St Vaast la Hougue, Manche, France | North Atlantic Ocean | 100 | 11.8 | Clarke & Wheeler, 1922 (as *Lithothamnium calcareum*) |
|  |  |  | Isle Glenan, Finistere, France | North Atlantic Ocean | 100 | 10.7 | Clarke & Wheeler, 1922 (as *Lithothamnium calcareum*) |
|  |  |  | Roscoff, Finistere, France | North Atlantic Ocean | 100 | 11.8 | Clarke & Wheeler, 1922 (as *Lithothamnium calcareum*) |
|  |  |  | Losini, middle Adriatic | Adriatic/Mediterrenean | 97 | 16 to 20 | Medakovic *et al*., 1995 |
|  |  | *repandum* | New Zeland | Pacific Ocean | 100 | 11.2 | Smith *et al*., 2012 |
|  | *Clathromorphum* | *compactum* | Torbay, Newfoundland | North Atlantic Ocean | 100 | 10.93 | Clarke & Wheeler, 1922 |
| Sporolitaceae | ***Heydrichia*** | *homalopasta* | New Zeland | Pacific Ocean | 100 | 12.8 | Smith *et al*., 2012 |
|  |  | *woelkerlingii* | New Zeland | Pacific Ocean | 100 | 14.5 | Smith *et al*., 2012 |
|  | ***Sporolithon*** | *durum* | New Zeland | Pacific Ocean | 98 to 100 | 12 to 15 | Smith *et al.*, 2012 |
|  |  | ***episporum*** | Point Toro, Isthmus of Panama | Caribbean | 100 | 13.09 | Clarke & Wheeler, 1922 |
|  |  |  | **Abrolhos Bank, Brazil** | **Southwest Atlantic Ocean** | **95.5** | **19.9** | **This study** |
|  |  | ***sp.*** | New Zeland | Pacific Ocean | 90 to 100 | 11 to 14 | Smith *et al.*, 2011 |
|  |  |  | **Rio de Janeiro, Brazil** | **Southwest Atlantic Ocean** | **81.7** | **23.8** | **This study (*)** |
|  |  | ***amadoi*** | **Fernando de Noronha Island, Brazil** | **Southwest Atlantic Ocean** | **97.8** | **24.5** | **This study (*)** |
|  |  | ***yoneshigueae*** | **Abrolhos Bank, Brazil** | **Southwest Atlantic Ocean** | **69.8** | **28.9** | **This study (*)** |

Figure S1:


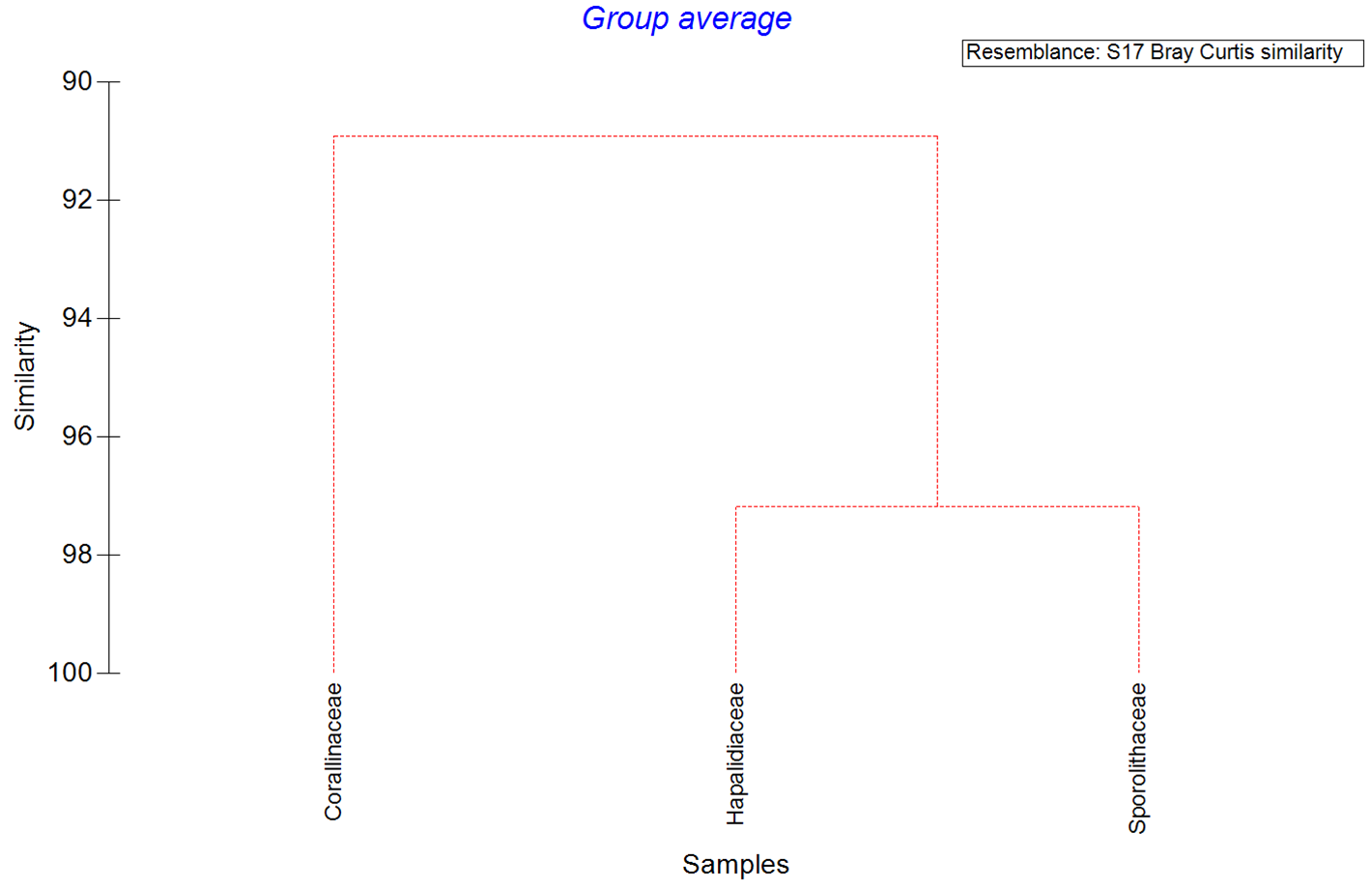


Figure S1: Cluster analysis of the mineralogy of the three coralline algae families. SIMPROF Test showed no difference between the mineralogy from Corallinaceae, Hapalidiaceae and Sporolithaceae.


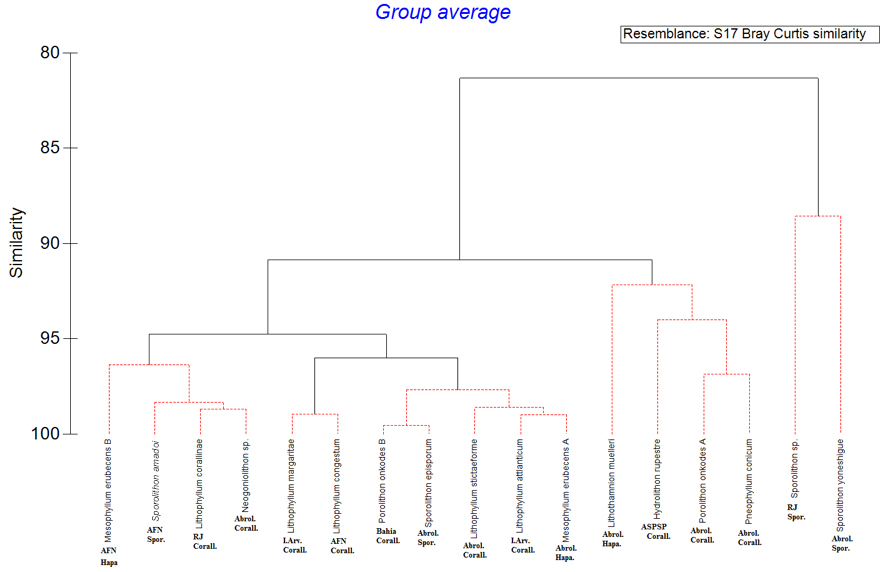
Figure S2

**A**

Figure S2: Cluster analysis of the mineralogy data from all the coralline algae species analyzed in this study. There were neither correlation between mineralogical data and coralline algae taxonomy nor collection site. Abbreviations: AFN – Archipelago of Fernando de Noronha; RJ – Rio de Janeiro; Abrol. –Abrolhos; I.Arv. – Island Arvoredo; ASPSP – Archipelago of São Pedro e São Paulo; Hapa. – Hapalidiaceae; Spor. – Sporolithaceae; Corall. – Corallinaceae.

Figure S3


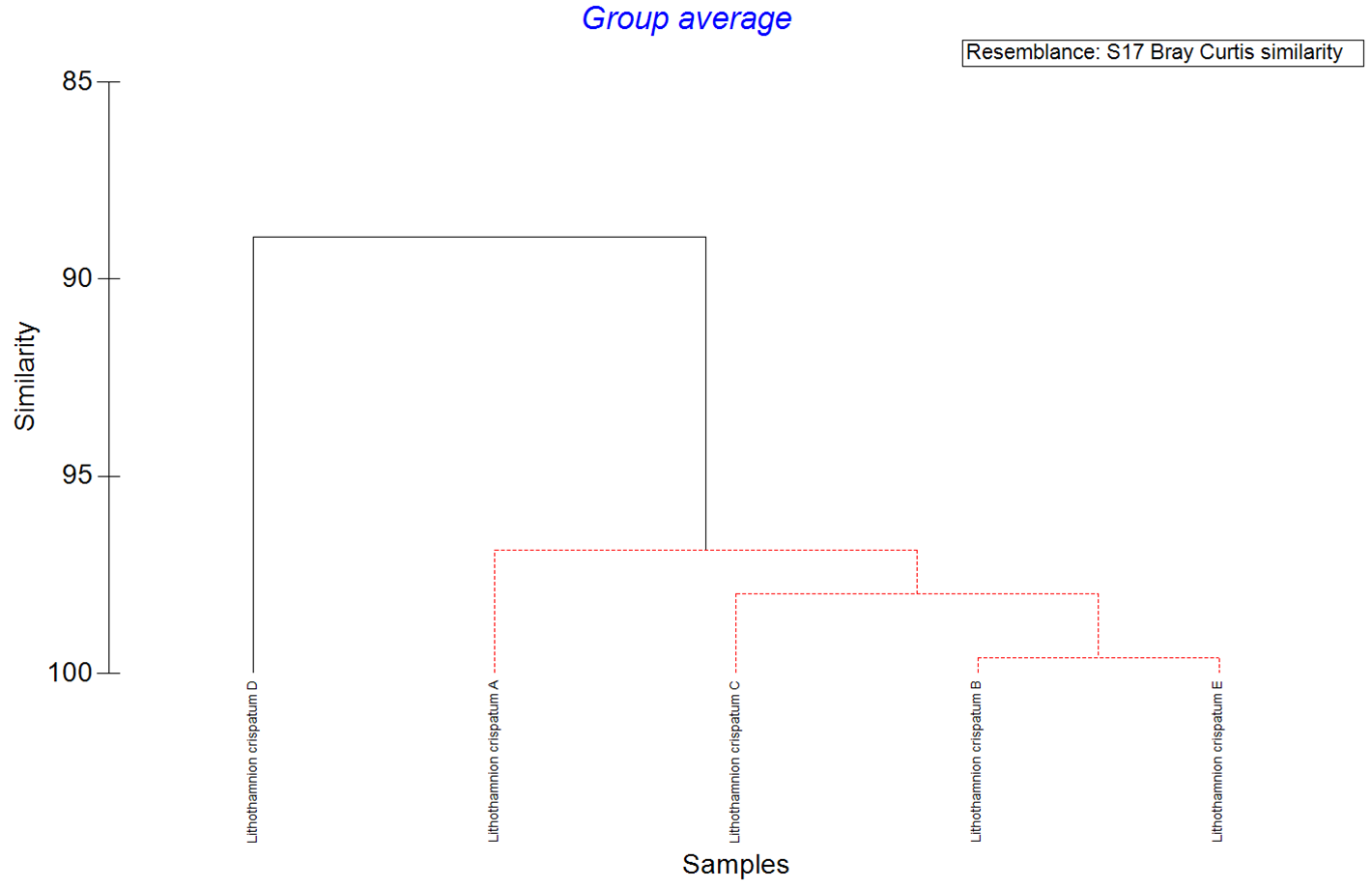


Figure S3: Cluster analysis of the mineralogical data from *Lithothamnion crispatum* samples collected in different sites: A – Amazonia; B – Trindade Island (ES); C – Espírito Santo; D – Abrolhos (BA); E – Salvador (BA). ES = Espírito Santo State and BA = Bahia State.

Figure S4


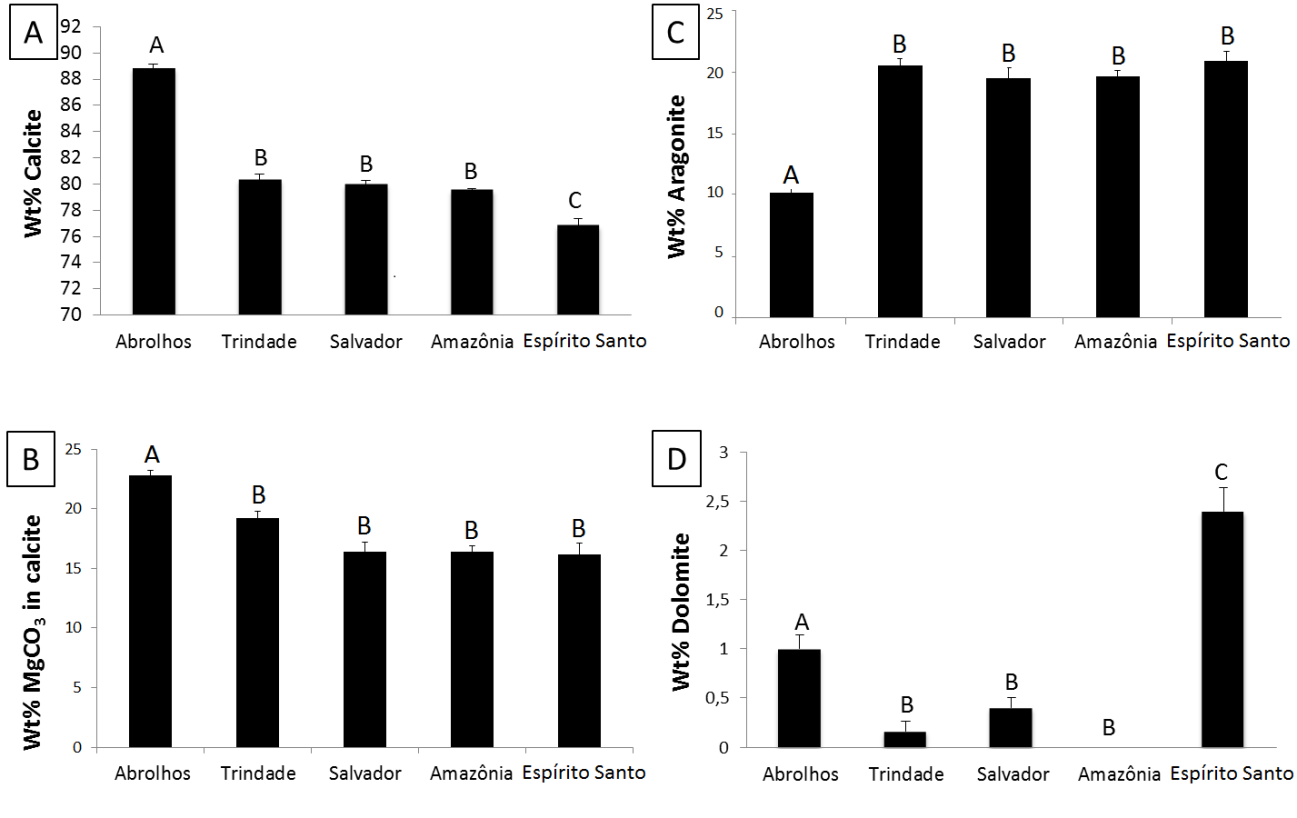


Figure S4: Mineralogical data from *Lithothamnion crispatum* collected in five different sampling sites: Abrolhos (BA), Trindade Island (ES), Salvador (BA), Amazonia and Espírito Santo (coordinates listed in Table S1). Specimens from Abrolhos presented a distinct pattern from other localities with more high-Mg calcite, less aragonite and the highest Mg substitution in calcite. ES = Espírito Santo State and BA = Bahia State.

Figure S5


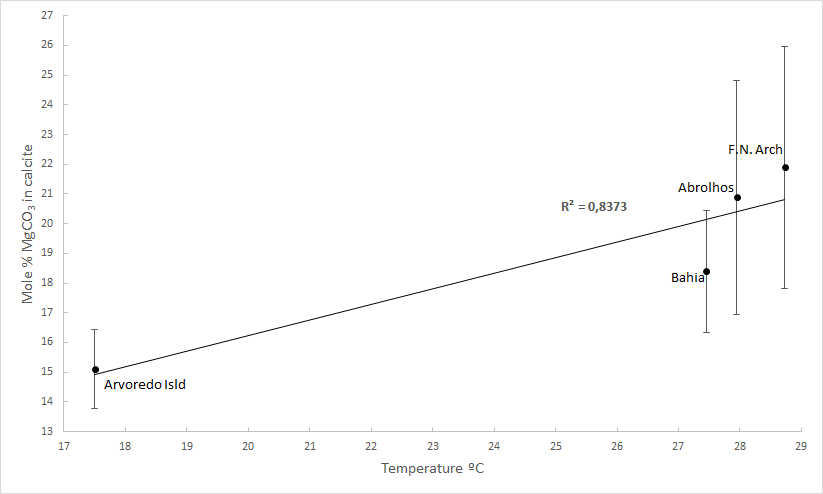
Figure S5: Relationship between Mole % MgCO_3_ in calcite and temperature from the locations with the highest sample size of coralline algae analyzed. Coefficient of determination from the linear regression (R^2^ = 0.8373) indicates that the increase of Mg substitution in calcite from Southwestern Ocean coralline algae is related to the increase of seawater temperature.
